# Precise Design and Deliberate Tuning of Turn-On Fluorescence in Tetraphenylpyrazine-Based Metal−Organic Frameworks

**DOI:** 10.34133/2022/9869510

**Published:** 2022-10-17

**Authors:** He-Qi Zheng, Lin Zhang, Mengting Lu, Xiaoyan Xiao, Yu Yang, Yuanjing Cui, Guodong Qian

**Affiliations:** State Key Laboratory of Silicon Materials, Cyrus Tang Center for Sensor Materials and Applications, School of Materials Science & Engineering, Zhejiang University, Hangzhou 310027, China

## Abstract

The manipulation on turn-on fluorescence in solid state materials attracts increasing interests owing to their widespread applications. Herein we report how the nonradiative pathways of tetraphenylpyrazine (TPP) units in metal−organic frameworks (MOFs) systems could be hindered through a topological design approach. Two MOFs single crystals of different topology were constructed via the solvothermal reaction of a TPP-based 4,4′,4^″^,4^‴^-(pyrazine-2,3,5,6-tetrayl) tetrabenzoic acid (H_4_TCPP) ligand and metal cations, and their mechanisms of formation have been explored. Compared with the innate low-frequency vibrational modes of flu net Tb-TCPP-1, such as phenyl ring torsions and pyrazine twists, Tb-TCPP-2 adopts a shp net, so the dihedral angle of pyrazine ring and phenyl arms is larger, and the center pyrazine ring in TPP unit is coplanar, which hinders the radiationless decay of TPP moieties in Tb-TCPP-2. Thereby Tb-TCPP-2 exhibits a larger blue-shifted fluorescence and a higher fluorescence quantum yield than Tb-TCPP-1, which is consistent with the reduced nonradiative pathways. Furthermore, Density functional theory (DFT) studies also confirmed aforementioned tunable turn-on fluorescence mechanism. Our work constructed TPP-type MOFs based on a deliberately topological design approach, and the precise design of turn-on fluorescence holds promise as a strategy for controlling nonradiative pathways.

## 1. Introduction

As an important branch of luminescent materials, organic fluorescent materials have gained extensive attention owing to their huge application prospects [1–3]. However, most organic fluorescent molecules undergo fluorescence quenching, particularly in the solid state, resulting in the decreased quantum yield (QY), which greatly interferes their development in the fields of fluorescent applications owing to the aggregation-caused quenching (ACQ) [4–7]. Mei et al., Mei et al., and Zhang et al. introduced aggregation-induced emission (AIE) to fluorescence fields, and the optical applications of the AIE effect have grown rapidly in recent years [6, 8, 9]. Recently, several examples of rigid metal−organic frameworks (MOFs) composed of tetraphenylethylene (TPE) derived ligands have shown that the turn-on fluorescence of AIE-type chromophores can be further enhanced due to coordination effect [10–16]. Despite these achievements, these MOFs were not full turn-on fluorescent materials due to their inherent low-frequency vibrational modes, mainly including the distortion of C=C double bond and the pore environment of MOFs that are sufficient to accommodate the free rotation of phenyl rings [12, 13, 17]. Meanwhile, the traditional reported rigid MOF systems are single state of turn-on fluorescence systems, which means the fluorescence properties of AIE cannot be adjusted [18, 19]. To address the challenge of hindering the innate low-frequency vibrational modes of AIE-type units, we turned to design and fabricate a class of tunable turn-on fluorescent materials.

Precise design and prediction of MOFs with different topologies can be easily achieved through reticular chemistry methods [20–24]. MOFs are built from ligands and metal ions/clusters, which means that the structure of MOFs can be adjusted by implementing a molecular building block (MBB) approach to obtain targeted polynuclear rare earth (RE) metal clusters, and tuning the geometry, length, and functional groups of the ligands [25–30]. In other words, the design and prediction of MOFs and topologies can be reached by changing RE metal clusters and ligands with well-defined geometries [31–33]. In particular, for adapting to a specific MOF structure, ligands often need to adjust their geometric characteristics, mainly including the twisting and torsion angles of adjacent rings and the relative positions of ligands [34, 35]. As a new type of AIEgens, tetraphenylpyrazine (TPP) molecules are highly twisted, which can behave variable fluorescence emissions when the twisting degree of the molecules is different [36, 37]. Yin et al. have studied that rotation restricted emission and antenna effect in TPP-based Gd-MOF that take advantage of these emissive features and promising detection functions [38]. However, this MOF system is single state of turn-on fluorescence systems. Thus, as a proof of the concept, MOFs constructed by this topological design approach could possess tunable structure and act as versatile platforms to explore properties of turn-on fluorescence. The tunability of structures through the topological design approach makes MOFs great candidates to: (i) study specific details of the twisting vibration of the pyrazine ring and the rotation of the phenyl ring in TPP units, (ii) control molecular conformations and nonradiative pathways of chromophores through changing the topology of MOFs, and (iii) explore the relationships of structures and luminescence in TPP-based MOFs.

With an eye toward hindering the nonradiative pathways to provide a turn-on fluorescent MOF and exploit it as a model, we have designed and synthesized two TPP-based tetrahedral carboxylate ligand H_4_TCPP based highly connected Tb-MOFs through a topological design approach. Compared with that in Tb-TCPP-1, the TPP unit in Tb-TCPP-2 shows significantly reduced pyrazine ring twist and phenyl ring rotation due to the rigidity of the MOF structure. The nonradiative pathway in Tb-TCPP-2 is hence hindered, resulting in a larger blue shift fluorescence and a higher fluorescence quantum yield. Furthermore, we coupled with DFT studies to propose that both the twisting of the pyrazine ring and the rotation of the phenyl rings can quench the fluorescence emission of the TPP unit. We expect that this topological design approach can contribute to an in-depth explanation of the turn-on fluorescence mechanism as well as provide a facile method to design tunable turn-on fluorescent materials.

## 2. Results

### 2.1. Structural and Topological Analyses

**Figure 1:**
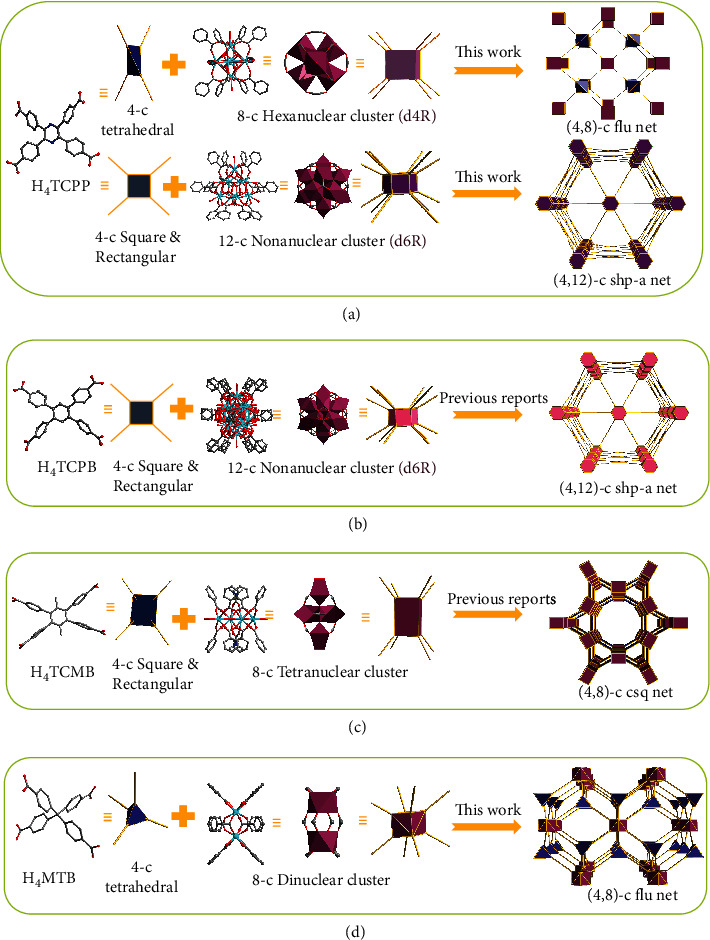
Schematic illustration of different topologies in MOFs based on various tetratopic carboxylate-based ligands: (a) employment of a 4-c ligand H_4_TCPP and an 8-connected RE_6_ clusters resulted in a (4,8)-c flu net; the self-assembly of 4-c ligand H_4_TCPP and 12-c RE_9_ clusters obtains (4,12)-c shp net; (b) the formation of a shp net by 12-c RE nonanuclear cluster and the tetratopic linker H_4_TCPB reported by Eddaoudi and coworkers; (c) the construction of (4,8)-c csq net from a 4-c ligand H_4_TCMB and 8-c RE_4_ cluster reported by Trikalitis and coworkers; (d) 8-c RE_2_ clusters are linked to tetratopic linker H_4_MTB generates a (4,8)-c flu net. Red: the RE clusters; black: C; red: O; blue: N.

**Figure 2:**
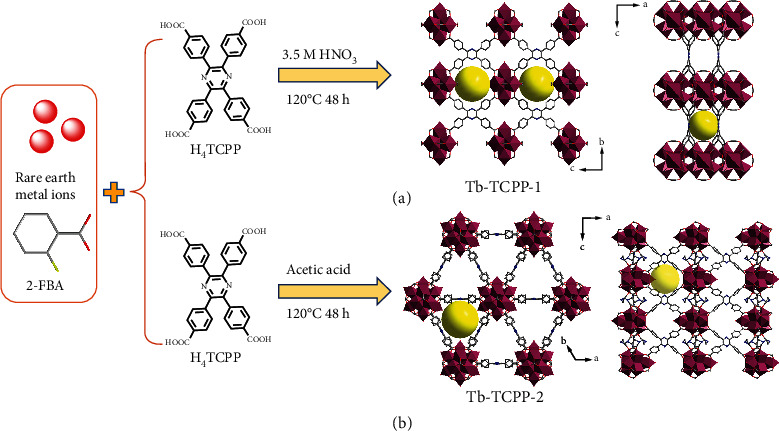
Schematic representation of the construction of (a) Tb-TCPP-1 and (b) Tb-TCPP-2. H atoms have been omitted and solvent molecules have been squeezed for clarity.

In this work, four tetratopic carboxylate-based ligands with various rigidities and symmetries were selected to discuss the structure and topology of different RE-MOFs. There are three topologies involved, namely, flu, shp, and csq net (Figure 1). Solvothermal reaction of H_4_TCPP ligand and RE^3+^ (RE: Eu^3+^, Tb^3+^, Y^3+^, Gd^3+^, Dy^3+^, Ho^3+^, Tm^3+^, Yb^3+^) in N,N′-dimethylformamide (DMF)/water/nitric acid (HNO_3_) mixture with the help of 2-fluorobenzoic acid (2-FBA) yielded octahedron-shaped Eu-TCPP-1 crystals. SCXRD analysis indicates that Eu-TCPP-1 adopts an orthorhombic space group of Fmmm (Table S1). The framework contains 4-c tetrahedral organic linker and 8-connected hexanuclear [Eu_6_(*μ*_3_-O)_8_(*μ*_3_-OH)_8_(Formate)_4_(−CO_2_)_8_] metal-cluster octahedral, which is similar to 8-c Zr_6_ clusters (Figure 1(a)). The (4,8)-connected Eu-TCPP-1 MOF framework forms with a flu topology, which is isoreticular to Zr-flu-SO_2_ [25], PCN-605 [27], Zr-CMOFs [39]. In this 3D porous framework, three quadrilateral-shaped channels are observed with the sizes of 12.2 Å × 10.4 Å, 16.6 Å × 6.9 Å, and 9.6 Å × 6.6 Å along the a, b, and c axis directions in the frameworks (Figure 2(a) and S1). The porosity of Eu-TCPP-1 was determined to be 56.4% using PLATON software analysis. The SCXRD studies revealed that Eu-TCPP-1 was formulated as [Eu_6_(*μ*_3_-O)_8_(*μ*_3_-OH)_8_(Formate)_4_(TCPP)_2_]·(solv)x. Since Eu-TCPP-1 and Tb-TCPP-1 are isomorphic, only Tb-TCPP-1 will be discussed later.

Interestingly, we found that when acetic acid was used instead of nitric acid, while 2-FBA/H_4_TCPP and RE(NO_3_)_3_·6H_2_O (RE: Tb^3+^ and Y^3+^) were consistent with the above, we obtained a shp net of hexagonal prism-shaped crystals (Figure 1(a)). Tb-TCPP-2 adopts a hexagonal space group of P6_3_/mmc (Table S1). The Tb-TCPP-2 consists of 4-c rectangular organic linker and 12-c nonanuclear clusters (Figure 1(a)). The nonanuclear cluster is anionic Tb_9_(*μ*_3_-O)_2_(*μ*_3_-OH)_12_(OH)_2_(H_2_O)_7_(O_2_C−)_12_]^7−^, and the resultant overall charge of the framework is balanced by (CH_3_)_2_NH^2+^. The (4,12)-connected Tb-TCPP-2 framework forms with a distinct square hexagonal prism shp topology, which is isoreticular to the reported NU-904 [40], PCN-223 [41], and Y-shp-MOF-5 [42]. The Tb-TCPP-2 structure forming 3D porous framework and the channel with a size of 11 Å along the c axis (Figure 2(b) and S2). It is a fact that Tb-TCPP-2 is similar to that of recently reported JNU-206-Tb [43], Y-TCPP [44], herein only Tb-TCPP-2 was studied for its luminescence behavior in the following sections.

As a comparison, substituting the pyrazine ring by a methyl group in the center would greatly increase the dihedral angles of methyl group and phenyl rings, thus a tetrahedral tetrakis(4-carboxyphenyl)methane (H_4_MTB) was chosen as ligand. Herein, the Eu-MTB crystals were gained in the solvothermal reaction between Eu^3+^ and H_4_MTB in DMF/H_2_O/HNO_3_ mixture, with 2-FBA as a modulator. SCXRD analysis indicates that Eu-MTB adopts a monoclinic space group of I2/m (Table S2). The (4,8)-connected Eu-MTB MOF framework forms with a flu topology, when combining of dinuclear [Eu_2_(*μ*_3_-OH)_8_(−CO_2_)_4_] cluster as MBBs and H_4_MTB ligands (Figure 1(d) and S4). The rhombic channels of Eu-MTB along the a, b, and c axes are as large as 12 Å × 17.1 Å, 11.6 Å × 9.4 Å, and 7.2 × 6.4 Å^2^ (Figures S3 and S4). The 3D framework of Eu-MTB has a formula of [Eu_2_(MTB)_2_]. The porosity of Eu-MTB was determined to be 58.6% based on the PLATON analysis. Note that our Tb-MTB with a structure similar to ionic Ln-MOF has been reported by Liu et al. and Lei et al. [45, 46].

By replacing the pyrazine group of TPP unit with a phenyl ring, the geometry of the tetratopic carboxylate-based ligand can be fixed on a square and rectangular planar. Then the 4,4′,4^″^,4^‴^-benzene-1,2,4,5-tetrayl-tetrabenzoic acid (H_4_TCPB) ligand was taken to discuss as a comparison. Interestingly, AbdulHalim et al. [42] recently have designed a MOF that contains the square and rectangular H_4_TCPB ligand and the 12-connected nonanuclear cluster [Y_9_(*μ*_3_-O)_2_(*μ*_3_-OH)_12_(OH)_2_(O_2_C−)_12_]^3−^ (Figure 1(b)). The (4, 12)-connected Y-shp-MOF-5 framework forms with a shp topology, which is isomorphic with aforementioned Tb-TCPP-2 and Y-TCPP [44].

To further investigate the effect of linker geometry and steric hindrance over confirmation of MOFs, two methyl groups are introduced into the central phenyl ring of the H_4_TCPB ligand, and then 1,2,4,5-tetrakis(4-carboxyphenyl)-3,6-dimethyl-benzene (H_4_TCMB) is chosen as ligand and discussed later. Angeli et al. [47] have reported the fabrication of Y-csq-MOF-1. The construction of (4,8)-connected Y-csq-MOF-1 from a 4-c rectangular linker H_4_TCMB and the 8-c tetranuclear cluster RE_4_(*μ*_3_-O)_2_(COO)_8_ (RE^3+^: Y^3+^, Tb^3+^, etc.). Y-csq-MOF-1 features a csq topology (Figure 1(c)), which is isoreticular to reported NU-1000 [48] and PCN-222 [49].

### 2.2. Formation Mechanism

**Figure 3:**
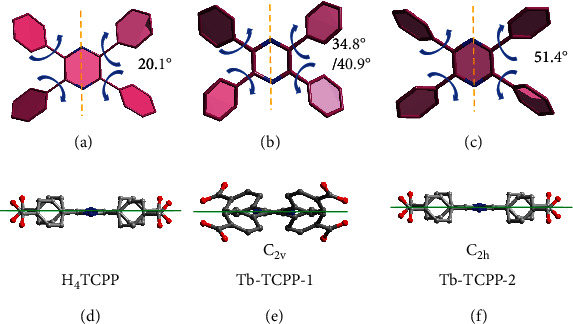
Tetratopic ligand conformations and twist angle between pyrazine core and phenyl arms in (a and d) free H_4_TCPP ligand, (b and e) Tb-TCPP-1, and (c and f) Tb-TCPP-2. The yellow line indicates the rotational axis.

To evaluate the formation mechanism of two MOFs based on H_4_TCPP ligands, the topologies of MOFs and the geometry of ligands were carefully analyzed. According to SCXRD, we note that in the (4,8)-connected flu net of Tb-TCPP-1, the TCPP^4-^ linker adopts a *C_2v_* symmetry to lower the symmetry of the linker. The TCPP^4-^ molecules twist to adapt to the Tb-TCPP-1 structure, which matches the 8-c Tb_6_ cluster in the flu net. Interestingly, we found that the center pyrazine core twisted into a nonplanar structure, so we can construct two planes, and the twist angle is 21.1° (Figure S5). Thus, the pyrazine ring and phenyl arms dihedral angle become 34.8° and 40.9°, respectively, in Tb-TCPP-1 from 20.1° of free H_4_TCPP [38, 50] (Figures 3(b) and 3(e)). On the contrary, so as to match the 12-c Tb_9_ cluster in the she net of Tb-TCPP-2, the two N atoms in pyrazine of TCPP^4-^ are coplanar to other carbon atoms, which are similar with the H_4_TCPB based shp net Y-shp-MOF-5 and the H_4_TCMB based csq net Y-csq-MOF-1. However, the linkers in Tb-TCPP-2 adopt C_2h_ symmetry with the phenyl arms rotating toward pyrazine core. Thus, after free H_4_TCPP self-assembles to form Tb-TCPP-2 MOF, the dihedral angle between the pyrazine core and phenyl arms would increase from 20.1° to 51.4° (Figures 3(c) and 3(f)). In fact, the dihedral angle increases from 40.9° to 51.4° in Tb-TCPP-1 and Tb-TCPP-2. As a comparison, Eu-MTB was constructed from tetrahedral H_4_MTB ligand shows the same D_2v_ symmetry and (4,8)-connected flu net (Figure 1(d) and S6). The four phenyl ring arms of the ligand are free to rotate around the central carbon atom.

### 2.3. Luminescent Properties

Fluorescence spectra of the ligand and all synthesized MOFs were also collected. The H_4_TCPP ligand is weakly emissive at ca. 465 nm, and the optimal excitation peak of the ligand was observed at 395 nm, which is presumed to be caused by the *π*-*π*^∗^ electronic transition of the ligand (Figure S15). While Tb-TCPP-1 only exhibited 417 nm emission bands when excited at 390 nm (Figure S17). The single microcrystal of Tb-TCPP-1 was found to emit bright blue light when excited at 365 nm by the fluorescence microscope (insets of Figure S17). Tb-TCPP-1 emits distinct blue light with a quantum yield (*Φ*_F_) of 6.87% (Table S3). The average lifetime of Tb-TCPP-1 is 0.5462 ns when excited by a 375 nm lamp (Table S3). Tb-TCPP-2 exhibits excellent performance of turn-on fluorescence when the H_4_TCPP ligands coordinate with metal clusters to form the MOF framework. Upon excitation of 365 nm, the crystals of as-synthesized Tb-TCPP-2 and Y-TCPP-2 exhibit the strong characteristic emission bands at 408 nm (Figures S18 and S19), which are assigned to the emission of H_4_TCPP ligand. Fluorescence microscopy revealed that Tb-TCPP-2 was a blue-purple single crystal (inset of Figure S18). It was calculated that the quantum yields (*Φ*_F_) of Tb-TCPP-2 is 15.91% (Table S3). The average lifetime of Tb-TCPP-2 is 0.6099 ns when excited by a 375 nm lamp (Table S3).

### 2.4. Tunable Turn-On Fluorescence

**Figure 4:**
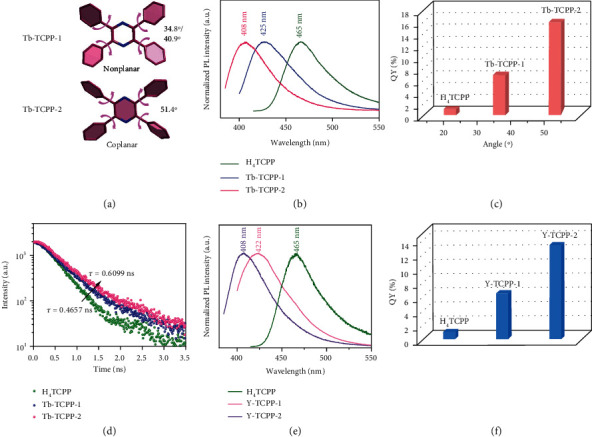
(a) Twist angle between pyrazine core and phenyl arms in Tb-TCPP-1 and Tb-TCPP-2. (b) Emission spectra, (c) QY and twist angles, and (d) lifetime of H_4_TCPP, Tb-TCPP-1, and Tb-TCPP-2 at room temperature (RT). (e) Emission spectra and (f) QY of H_4_TCPP, Y-TCPP-1, and Y-TCPP-2 at room temperature (RT).

For instance, tetrakispyrazine (TPP), a new kind of AIEgen reported by Tang et al., would possess the AIE effect owing to typical restriction of intramolecular motion (RIM) mechanism [36]. Then we carried out the test including the fluorescence and the quantum yields of H_4_TCPP ligand in mixed solvents (For specific analysis, see Supporting Information, Figure S24, Table S3). As aforementioned, for TPP chromophores in dilute solution, the phenyl rings connected to pyrazine ring through single bonds can rotate freely, acting as a relaxation channel via nonradiative decay. Unlike the above, in our MOF system, the rotatable TPP unit in H_4_TCPP is fixed with metal clusters to enhance the emission due to the coordination effect [11]. Moreover, as aforementioned, the peak emission shifts from 465 nm for H_4_TCPP to 425 nm and 408 nm for Tb-TCPP-1 and Tb-TCPP-2, separately. About 40 nm and 57 nm blueshifts were observed for Tb-TCPP-1 and Tb-TCPP-2, respectively (Figure 4(b)). The same excitation spectra are observed from the Tb-TCPP-1 and Tb-TCPP-2 systems (Figure S25). The planar structure of H_4_TCPP twists to fit the rigid MOF, which is the additional evidence for coordination and is responsible for blue-shifted emission rather than simple irregular aggregation in the solid state (Figure 4(b)). Apart from the blue-shifted emission, we can also find that the fluorescence has been significantly enhanced after the TPP chromophore is rigidified in MOFs (Figure S26). As to H_4_TCPP, Tb-TCPP-1, and Tb-TCPP-2, the twist angles aforementioned were 20.1°, 40.9°, and 51.4°, respectively, which are consistent with their fluorescence quantum efficiencies (1.03%, 6.87%, and 15.91%, respectively) (Figures 4(a) and 4(c)). Compared to the ligand, the MOFs exhibited 6.7- to 15.4-folds high luminescence quantum yields. These results indicate that the larger the dihedral angle, the more enhanced the fluorescence quantum efficiency, which related to the intramolecular rotation mechanism. Furthermore, fluorescence decay studies showed that the average lifetimes of Tb-TCPP-1 and Tb-TCPP-2 in solid state were 0.5462 ns and 0.6099 ns, separately (Figure 4(d) and Table S3). For the first time, we found that the center pyrazine ring of Tb-TCPP-1 twisted into a nonplanar structure based on SCXRD data (the dihedral angle is about 21.1°), while the center pyrazine ring of Tb-TCPP-2 is a planar structure (Figure S5). We guess that diminution of the pyrazine ring twisting angle will hinder the radiationless decay of TPP moieties in the locked state, and then enhance the fluorescence of Tb-TCPP-2. As mentioned above, the trends in blue shifts, quantum yields, and the fluorescence lifetimes of materials are uniform and related to the degree of the dihedral angle, confirming that topological design approach is an effective strategy to construct tunable turn-on fluorescent materials. Apart from the enhanced quantum yield of MOFs, a temperature-dependent fluorescence behavior was also observed. Taken Tb-TCPP-2 as an example, generally, as temperature decreases from 318 K toward 203 K, the fluorescence intensity gradually increases due to the suppressed thermal quenching via relaxation pathways (Figure S27).

The turn-on fluorescence can be further extended to other H_4_TCPP based isomorphic RE-MOFs. Since some RE metal ions have characteristic luminescence, in order to exclude the influence of RE metal ions, we choose Y-based MOFs as a comparison for further discussion. As can be seen from Figure 4(e), the peak emission shift from 465 nm for H_4_TCPP to 422 nm and 408 nm for Y-TCPP-1 and Y-TCPP-2. About 43 nm and 57 nm blueshifts were observed for Y-TCPP-1 and Y-TCPP-2, separately. Since Y-TCPP-1 and Y-TCPP-2 are isomorphism of Tb-TCPP-1 and Tb-TCPP-2, respectively, their dihedral angles are same, respectively, which are consistent with their fluorescence quantum efficiencies (6.47% and 13.28%, separately) (Figure 4(f) and Table S3). Furthermore, the fluorescence decay profiles showed that the average lifetimes of the Y-TCPP-1 and Y-TCPP-2 in solid state are 0.7117 ns and 0.8484 ns, respectively, which are consistent with the quantum efficiency (Figure S28 and Table S3). As expected, the difference, including the blue shifts, quantum yields, and lifetimes, between Y-TCPP-1 and Y-TCPP-2 is almost identical to those of Tb-TCPP-1 and Tb-TCPP-2. Therefore, the RE metal ions had little influence on the emission behaviors of the RE-MOFs, while the restriction of intramolecular motion of TPP units played a key role.

### 2.5. Theory Computations

**Figure 5:**
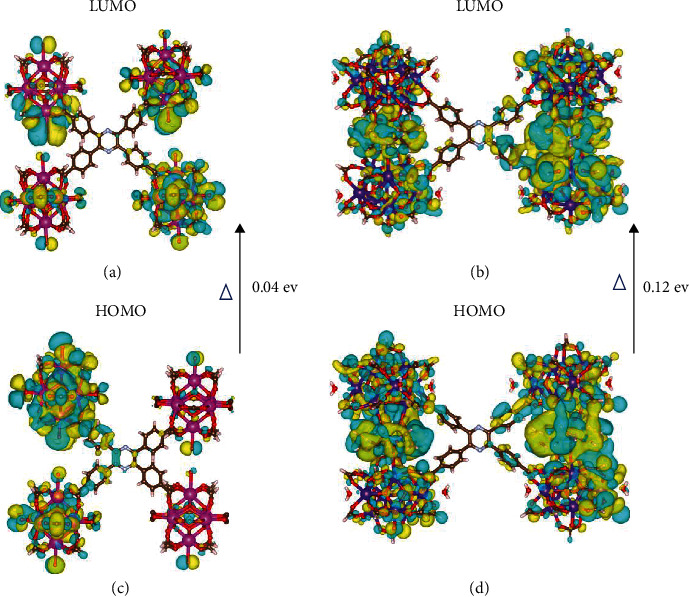
Isodensity plots of the HOMO and LUMO for H_4_TCPP in (a and c) Tb-TCPP-1 and (b and d) Tb-TCPP-2 conformation based on DFT calculations.

To provide microscopic insight into the phenyl ring and pyrazine ring dynamics in TPP based MOFs, the electronic structures of Tb-MOFs were optimized via the DFT calculations method (Figure 5). For Tb-TCPP-1 and Tb-TCPP-2, the main adsorption bands are located at around 390 nm and 365 nm, respectively, which correspond to the HOMO and LUMO transitions (Figure 5, S25 and S29). As discussed above, compared with the free conformation of H_4_TCPP, the dihedral angles of the rigid MOFs are positively correlated with the fluorescence of MOFs. For metal complexes, the larger dihedral angle leads to the destruction of the delocalized conjugated system of the ligands, thus widening the energy gap between HOMO and LUMO. We believe that this decomposition of the conjugated system is the main reason for the large blue shift in the UV-vis absorption and emission bands of MOFs [14, 38]. As can be seen in Figure 5, the band gaps of the Tb-TCPP-1 and Tb-TCPP-2 frameworks are 0.04 eV and 0.12 eV, respectively. Therefore, the fluorescence emission of Tb-TCPP-2 has a larger blue shift than Tb-TCPP-1 (Figure 4). Apart from the blue shifts, the rigid MOFs greatly restrict the intramolecular motion of TPP units, hindering the radiationless pathway of TPP units, and substantially increasing the fluorescence quantum yield of MOFs [51].

**Figure 6:**
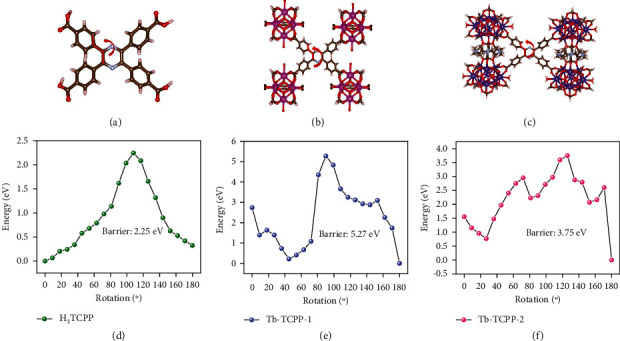
Theoretical calculations: PESs for the single bond of pyrazine ring and phenyl rings in models of TPP. Structures of truncated RE cluster-capped models and flipping energy barrier of (a and d) H_4_TCPP and two TPP units in the constructed (b and e) Tb-TCPP-1 and (c and f) Tb-TCPP-2. The hydrogen atoms are omitted in the depicted models for clarity.

To estimate the rotational energy barrier (*E*_a_) for ring flipping, we thus modeled the potential energy surfaces (PESs) of TCPP^4-^ bound by four Tb clusters [12, 51, 52]. Notably, the *E*_a_ value was obtained over modeling the PESs by varying the phenyl-phenyl-pyrazine dihedral angle from 0 to 180° and 180 to 0° with an interval of 10°. For instance, we found that the activation barrier for the unrestricted H_4_TCPP ligand in the solid phase is 2.25 eV (Figures 6(a) and 6(d)). Two minima were observed at 45° and 180° for Tb-TCPP-1, and two minima were observed at 30° and 180° for Tb-TCPP-2, indicating the presence of stable conformations. The rotation barriers were calculated to be 5.27 eV in Tb-TCPP-1 and 3.75 eV in Tb-TCPP-2, which were approximately 3.02 eV and 1.50 eV higher than that of H_4_TCPP, implying the restriction of the phenyl ring rotation in TPP units are thus partially restricted and the radiationless decay is partially blocked, which explains why our MOFs are turn-on fluorescence systems (Figure 6) [13, 51]. This verified that the restriction of intramolecular rotation mechanism is indeed the main reason for the turn-on fluorescence of the TPP units in our MOFs system [36, 53].

Since phenyl ring torsion has a higher contribution to the nonradiative decay of the excited state then the C=C bond twist in TPE derivatives has been addressed before [54]. According to Tb-TCPP-2 (*E*_a_, 3.75 eV) required for a low-barrier phenyl ring torsion then Tb-TCPP-1 (*E*_a_, 5.27 eV). Therefore, Tb-TCPP-1 allows the TPP unit to have the maximum fluorescent quantum yield, while Tb-TCPP-2 has the minimum in theory. However, our results were inconsistent with this observation and allow us to establish new explanations about it. After metal clusters locked the H_4_TCPP ligand, the intramolecular vibration of TPP units (mainly the twisting of pyrazine ring) becomes restricted, which is in some like the C=C bond twist in TPE molecular [6]. Previously, the rigid coplanar structures in the solid state can increase the effective conjugation length and suppress the nonradiative decay and improved photon absorption and emission properties [55–57]. Since the pyrazine ring of Tb-TCPP-2 is rigid and coplanar, the low-frequency vibrational is hindered, the radiationless pathway is blocked, and the radiative decay channel is opened, which enhances the emission of Tb-TCPP-2. As to Tb-TCPP-1, the pyrazine ring in TPP unit twisting to nonplanar, the extent of intramolecular twisting of Tb-TCPP-1 is further increased compared to Tb-TCPP-2. We believe that the difference of the vibrational energy of two MOFs is the main factor (rate-determining step) affecting the fluorescence of Tb-TCPP-1, while the rotational energy barrier is the secondary factor, since phenyl rings rotation is affected by the twist of the pyrazine ring [54, 58]. Therefore, the twist angle of the pyrazine ring of the TPP unit in Tb-TCPP-1 increases, resulting in enhanced nonradiative decay, which makes Tb-TCPP-1 exhibits lower fluorescence intensity, quantum yield, and lifetime. Those results mentioned above show the twisting of the pyrazine ring and the rotation of the phenyl rings can quench the emission in TPP units, which are similar to the dynamics and mechanism of AIE in TPE-based MOF system [13]. Since AIE is an important photophysical effect in nature, the study of the AIE mechanism and the development of tunable turn-on fluorescence materials based on TPP AIE-MOFs are important for applications.

## 3. Discussion

In summary, guided by a topological design approach, two new and fascinating highly connected MOFs were synthesized, and the formation mechanisms were explored when combined with a series of TPP-based ligand and RE metal cations. With the existence of an AIE H_4_TCPP ligand, we designed and obtained two novel MOFs, Tb-TCPP-1 and Tb-TCPP-2, that display a (4,8)-coordinated flu net and a (4,12)-coordinated shp net, based on hexanuclear Tb_6_ clusters and nonanuclear Tb_9_ clusters, respectively. Interestingly, due to a variety of the torsion of the phenyl rings and the pyrazine rings twist in TPP units, Tb-TCPP-1 and Tb-TCPP-2 were endowed with the diversity of the coordination effect. Since the emission blueshifts variously and quantum yield enhanced variously in Tb-TCPP-1 and Tb-TCPP-2, which are complementary to AIE. Furthermore, we coupled with DFT studies to propose that both the twisting of pyrazine core and the rotation of the phenyl rings can quench the emission in TPP units. Our work constructed TPP-type MOFs based on deliberately topological design approach, and the precise design and deliberate turning of locked conformation provides a strategy for hindering nonradiative pathways.

## 4. Materials and Methods

### 4.1. Materials and Chemicals

RE(NO_3_)_3_·6H_2_O (RE: Eu^3+^, Tb^3+^, Y^3+^, Gd^3+^, Dy^3+^, Sm^3+^, Ho^3+^, Er^3+^, Tm^3+^, Yb^3+^, Nd^3+^, Lu^3+^, La^3+^, Pr^3+^), 2-FBA, and other reagents were bought from Energy Chemical. H_4_TCPP and H_4_MTB ligands were purchased from Yanshen Technology Co., Ltd. All reagents and solvents were purchased from commercial sources.

### 4.2. Synthesis of Eu-TCPP-1

The Eu-TCPP-1 microcrystals were synthesized with the classical solvothermal method. Eu(NO_3_)_3_·6H_2_O (41 mg, 0.092 mmol), H_4_TCPP (6.16 mg, 0.011 mmol), 2-FBA (642.1 mg, 4.58 mmol), DMF (3.0 mL), H_2_O (0.5 mL), and HNO_3_ (0.15 mL, 3.5 M in H_2_O) were mixed in a Teflon-lined stainless steel vessel (21 mL) at room temperature, and then heated to 120 °C for 48 h. Colorless octahedron-shaped crystals were obtained at room temperature. Calc (%) for [Eu_6_(*μ*_3_-O)_8_(*μ*_3_-OH)_8_(Formate)_4_(TCPP)_2_]·(solv)x (C_140_H_64_Eu_12_N_8_O_68_): C, 24.70; H, 2.55; N, 2.41; O, 14.75. Found: C, 25.30; H, 2.40; N, 2.47; O, 14.87. Tb-TCPP-1 and RE-TCPP-1 (RE: Y^3+^, Yb^3+^, Tm^3+^, Dy^3+^, Ho^3+^) were synthesized similarly to Eu-TCPP-1 except for the use of RE(NO_3_)_3_·6H_2_O.

### 4.3. Synthesis of Tb-TCPP-2

The Tb-TCPP-2 microcrystals were synthesized with the classical solvothermal method. To a 20 mL glass vial containing Tb(NO_3_)_3_·6H_2_O (14.8 mg, 0.034 mmol), H_4_TCPP (5.0 mg, 0.009 mmol) and 2-FBA (300 mg, 2.14 mmol) dissolved in DMF (3.0 mL) and acetic acid (1.0 mL). The vial was heated to 120 °C for 48 h. Colorless regular hexagonal prism-shaped crystals were obtained. Calcd (%) for |DMA|_7_[Tb_9_(*μ*_3_-O)_2_(*μ*_3_-OH)_12_(OH)_2_(H_2_O)_7_(TCPP)_3_]·(solv)x (C_116_H_81_N_13_O_47_Tb_9_): C, 39.30; H, 3.68; N, 3.99; O, 18.31. Found: C, 40.83; H, 3.70; N, 3.97; O, 18.40. Y-TCPP-2 was synthesized similarly to Tb-TCPP-2 except for the use of Y(NO_3_)_3_·6H_2_O.

### 4.4. Synthesis of Eu-MTB

The Eu-MTB microcrystals were synthesized with the classical solvothermal method. A mixture of Eu(NO_3_)_3_·6H_2_O (18 mg, 0.040 mmol), H_4_MTB (27.5 mg, 0.055 mmol), 2-FBA (321 mg, 2.29 mmol), DMF (2.0 mL), H_2_O (250 *μ*L), and HNO_3_ (75 *μ*L, 3.5 M in H_2_O) were added to a 20 mL glass vial, and then heated to 110 °C for 24 h. Colorless block cuboid crystals were collected. Calcd (%) for [Eu_2_(MTB)_2_] (C_58_H_32_Eu_2_O_16_): C, 49.60; H, 4.47; N, 4.51, O, 23.64. Found: C, 49.73; H, 4.75; N, 4.52, O, 23.52. RE-MTB (RE: Tb^3+^, Gd^3+^, Dy^3+^, Ho^3+^, Er^3+^, Tm^3+^, Yb^3+^, Nd^3+^, Lu^3+^, Sm^3+^, La^3+^, Pr^3+^) were synthesized similarly to Eu-MTB except for the use of RE(NO_3_)_3_·6H_2_O.

## Data Availability

All data needed in the paper include more detailed experimental methods are available in Supplementary section.

## Supplementary Materials

Supplementary Materials Table S1: crystal data of Eu-TCPP-1 and Tb-TCPP-2. Table S2: crystal data of Eu-MTB. Table S3: photophysical parameters including Quantum yield (QY, *Φ*F) and average lifetime of ligands and RE-MOFs. Figure S1: three type of channels in Eu-TCPP-1. Figure S2: three type of channels in Tb-TCPP-2. Figure S3: three type of channels in Eu-MTB. Figure S4: schematic representation of the construction of Eu-MTB. Figure S5: (a) the average of dihedral of P1-P2 plane is the twist pyrazine rings of the H_4_TCPP in Tb-TCPP-1; (b) P1-P2 is the average plane of the central pyrazine rings, P3 is the average plane of the phenyl rings adjacent to pyrazine rings of the H_4_TCPP in Tb-TCPP-1 optimized conformation, and the dihedral of P1-P3 and P2-P3 are 34.8° and 40.9°, respectively; (c) P1 is the average plane of the central pyrazine rings. P2 is the average plane of the phenyl rings adjacent to pyrazine rings. Figure S6: conformations of the ligands in Eu-MTB. (a and b) The free tetrahedral H_4_MTB ligand. (c and d) Conformation of the tetrahedral ligand in the flu net framework. Black: C; red: O. Figure S7: PXRD patterns of synthesized (a) Tb-TCPP-1 and Eu-TCPP-1 and (b) Tb-TCPP-2 and Y-TCPP-2. Figure S8: PXRD patterns of RE-TCPP-1 synthesized with different RE^3+^ salts. Figure S9: PXRD patterns of synthesized Eu-MTB and Tb-MTB. Figure S10: PXRD patterns of RE-MTB synthesized with different RE^3+^ salts. Figure S11: SEM images of (a) Eu-TCPP-1 and (b) Tb-TCPP-2 and Eu-MTB. Figure S12: TGA curves for Eu-TCPP-1 under nitrogen. Figure S13: TGA curves for Tb-TCPP-2 under nitrogen. Figure S14: TGA curves for Eu-MTB under nitrogen. Figure S15: luminescent spectra of H_4_TCPP powder. Figure S16: emission spectra and crystal photographs of Eu-TCPP-1 excited at 377 nm. Figure 17: emission spectra and crystal photographs of (a) Tb-TCPP-1 excited at 390 nm. Scale bar, 20 *μ*m, 50 *μ*m. Figure 18: emission spectra and crystal photographs of Tb-TCPP-2. Figure S19: emission spectra and crystal photographs of Y-TCPP-2. Figure S20: emission spectra and crystal photographs of Y-TCPP-1 excited at 380 nm. Figure S21: emission spectra and crystal photographs of (a) Eu-MTB and (b) Tb-MTB excited at 335 nm. Figure S22: emission spectra H_4_MTB powder. Figure S23: emission spectra of Gd-MTB. Figure S24: (a and b) emission spectra and plot of maximum PL intensity of ligand H_4_TCPP versus H_2_O fraction in DMF/H_2_O mixtures. Figure S25: the excitation and emission spectra of Tb-TCPP-1, Tb-TCPP-2, and H_4_TCPP at room temperature (RT). Figure S26: the emission spectra of H_4_TCPP, Tb-TCPP-1, and Tb-TCPP-2 in solid state. Figure 27: temperature-dependent emission spectrum of Tb-TCPP-2 cooled with liquid nitrogen. *Λ*ex = 365 nm. Figure S28: the lifetime of H_4_ Y-TCPP-1, and Y-TCPP-2 excited at 375 nm. Figure S29: contour plots of the HOMO and LUMO for H_4_TCPP, Tb-TCPP-1, and Tb-TCPP-2. Figure S30: fluorescence lifetime measurements for a powder sample of H_4_TCPP using an excitation of 375 nm and an emission of 465 nm. Figure S31: fluorescence lifetime measurements for a powder sample of Tb-TCPP-1 using an excitation of 375 nm and an emission of 415 nm. Figure S32: fluorescence lifetime measurements for a powder sample of Tb-TCPP-2 using an excitation of 375 nm and an emission of 408 nm. Figure S33: fluorescence lifetime measurements for a powder sample of Y-TCPP-1 using an excitation of 375 nm and an emission of 422 nm. Figure S34: fluorescence lifetime measurements for a powder sample of Y-TCPP-2 using an excitation of 375 nm and an emission of 408 nm.
