# Supplementary material for: Precise Design and Deliberate Tuning of Turn-On Fluorescence in Tetraphenylpyrazine-Based Metal−Organic Frameworks
Source: Research (Wash D C). 2022 Oct 17;2022:9869510. doi: 10.34133/2022/9869510 (PMC9609278; doi:10.34133/2022/9869510)
Supplement: Supplementary Materials — Table S1: crystal data of Eu-TCPP-1 and Tb-TCPP-2. Table S2: crystal data of Eu-MTB. Table S3: photophysical parameters including Quantum yield (QY, ΦF) and average lifetime of ligands and RE-MOFs. Figure S1: three type of channels in Eu-TCPP-1. Figure S2: three type of channels in Tb-TCPP-2. Figure S3: three type of channels in Eu-MTB. Figure S4: schematic representation of the construction of Eu-MTB. Figure S5: (a) the average of dihedral of P1-P2 plane is the twist pyrazine rings of the H4TCPP in Tb-TCPP-1; (b) P1-P2 is the average plane of the central pyrazine rings, P3 is the average plane of the phenyl rings adjacent to pyrazine rings of the H4TCPP in Tb-TCPP-1 optimized conformation, and the dihedral of P1-P3 and P2-P3 are 34.8° and 40.9°, respectively; (c) P1 is the average plane of the central pyrazine rings. P2 is the average plane of the phenyl rings adjacent to pyrazine rings. Figure S6: conformations of the ligands in Eu-MTB. (a and b) The free tetrahedral H4MTB ligand. (c and d) Conformation of the tetrahedral ligand in the flu net framework. Black: C; red: O. Figure S7: PXRD patterns of synthesized (a) Tb-TCPP-1 and Eu-TCPP-1 and (b) Tb-TCPP-2 and Y-TCPP-2. Figure S8: PXRD patterns of RE-TCPP-1 synthesized with different RE3+ salts. Figure S9: PXRD patterns of synthesized Eu-MTB and Tb-MTB. Figure S10: PXRD patterns of RE-MTB synthesized with different RE3+ salts. Figure S11: SEM images of (a) Eu-TCPP-1 and (b) Tb-TCPP-2 and Eu-MTB. Figure S12: TGA curves for Eu-TCPP-1 under nitrogen. Figure S13: TGA curves for Tb-TCPP-2 under nitrogen. Figure S14: TGA curves for Eu-MTB under nitrogen. Figure S15: luminescent spectra of H4TCPP powder. Figure S16: emission spectra and crystal photographs of Eu-TCPP-1 excited at 377 nm. Figure 17: emission spectra and crystal photographs of (a) Tb-TCPP-1 excited at 390 nm. Scale bar, 20 μm, 50 μm. Figure 18: emission spectra and crystal photographs of Tb-TCPP-2. Figure S19: emission spectra and crystal photographs of Y- [file 9869510.f1.docx]

Supporting Information

**Precise Design and Deliberate Tuning of Turn-On Fluorescence in Tetraphenylpyrazine-Based Metal−Organic Frameworks**

He-Qi Zheng, Lin Zhang, Mengting Lu, Xiaoyan Xiao, Yu Yang, Yuanjing Cui*, and Guodong Qian*

State Key Laboratory of Silicon Materials, Cyrus Tang Center for Sensor Materials and Applications, School of Materials Science & Engineering, Zhejiang University, Hangzhou 310027, China.

Yuanjing Cui, E-mail: cuiyj@zju.edu.cn; Guodong Qian, E-mail: gdqian@zju.edu.cn

**1-Experimental Section/Methods**

***1.1 Measurement and analysis***

Powder X-ray diffraction (PXRD) patterns of samples were recorded on an X’Pert Pro X-ray diffractometer. The C, H, O and N elemental analyses (EA) were measured using an EA1112 microelemental analyzer. The thermogravimetric curves from 30 to 800 °C in N_2_ atmosphere were performed on a Netszch TGA 209 F3 thermogravimeter with a heating rate of 5 °C min^−1^. The scanning electron microscopy (SEM) images were measured using a field-emission scanning electron microscopy (FE-SEM, Hitachi S4800). The photoluminescent (PL) spectra at room temperature were studied by a Hitachi F4600 fluorescence spectrometer. The luminescence decay curves were measured by an Edinburgh Instrument F920 spectrometer. The luminescence decay curves of ligand and MOFs were measured using a laser (375 nm) as the excited source. The overall quantum yields of the solid-state and solution samples were determined by an absolute method using an integrating sphere on an Edinburgh Instrument FLS980 spectrometer. The solid-state fluorescence spectra of MOFs were measured by a Hitachi F4600 fluorescence spectrometer and Xenon lamp serves as an excitation light source.

***1.2 Single-Crystal X-ray Crystallography***

The crystallographic measurement and structure determination of RE-MOFs were similar to our previous literature. The single-crystal X-ray diffraction (SCXRD) collection was taken on a Bruker APEX-II diffractometer, and the structure was determined by direct methods and refined with the SHELX-2014 program package. The SQUEEZE program of PLATON was used to remove guests from the disordered species. Topology analysis of MOFs was performed using the TOPOS 4.0 program package. Crystallographic data collection and refinement results are summarized in Table S1. **CCDC 2175037** and **2175039** contains the supplementary crystallographic data of RE-MOFs (Eu-MTB, Eu-TCPP-1), which can be obtained from the authors or the Cambridge Crystallographic Data Centre via www. ccdc.cam.ac.uk/data_request/cif.

**1.3 Density** **Functional** **Theory (DFT) Computation Details.**

All the DFT calculations were carried out using the Vienna ab initio simulation (VASP5.4.4) code[1]. The exchange-correlation is simulated with PBE functional and the ion-electron interactions were described by the PAW method[2, 3]. The Monkhorst-Pack-grid-mesh-based Brillouin zone k-points are set as 1×1×1 for all periodic structure with the cutoff energy of 400 eV. The convergence criteria are set as 0.01 eV A^-1^ and 10 4 eV in force and energy, respectively. The connection between MOF units is saturated with H atom. A cube with size of 35 ×35 ×35 Å is employed to accommodate the free standing MOF and to avoid interlayer interference meanwhile.

**2- Characterization** **data**

**2.1-Tables**

**Table S1.** Crystal data of Eu-TCPP-1 and Tb-TCPP-2.

| Compunds | **Tb-TCPP-2** | | **Eu-TCPP-1** | |  |
| --- | --- | --- | --- | --- | --- |
| CCDC |  | | 2175039 | |  |
| Empirical formula | | C_116_H_81_N_13_O_47_Tb_9_ | | C_140_H_64_Eu_12_N_8_O_68_ | |
| Formula weight | 3839.37 | | 4769.51 | |  |
| Temperature/K | 297.15 | | 273.15 | |  |
| Crystal system | hexagonal | | orthorhombic | |  |
| Space group | P6_3_/mmc | | Fmmm | |  |
| a/Å | 21.7666(3) | | 13.939(4) | |  |
| b/Å | 21.7666(3) | | 29.996(6) | |  |
| c/Å | 25.4614(6) | | 32.648(7) | |  |
| α/° | 90 | | 90 | |  |
| β/° | 90 | | 90 | |  |
| γ/° | 120 | | 90 | |  |
| Volume/Å^3^ | 10447.1(4) | | 13651(6) | |  |
| Z | 1.99992 | | 2 | |  |
| ρ_calc_g/cm^3^ | 1.220 | | 1.160 | |  |
| μ/mm^‑1^ | 15.111 | | 2.766 | |  |
| F(000) | 3658.0 | | 4520.0 | |  |
| Crystal size/mm^3^ | 0.2 × 0.1 × 0.1 | | 0.1 × 0.1 × 0.08 | |  |
| Radiation | CuKα (λ = 1.54178) | | MoKα (λ = 0.71073) | |  |
| 2Θ range for data collection/° | 4.688 to 136.568 | | 4.94 to 58.234 | |  |
| Index ranges | -26 ≤ h ≤ 26, -26 ≤ k ≤ 25, -30 ≤ l ≤ 27 | | -18 ≤ h ≤ 18, -39 ≤ k ≤ 40, -43 ≤ l ≤ 40 | |  |
| Reflections collected | 102583 | | 20607 | |  |
| Independent reflections | 3560 [R_int_ = 0.0664, R_sigma_ = 0.0201] | | 4774 [R_int_ = 0.0863, R_sigma_ = 0.0895] | |  |
| Data/restraints/parameters | 3560/8/55 | | 4774/46/147 | |  |
| Goodness-of-fit on F^2^ | 1.245 | | 1.018 | |  |
| Final R indexes [I>=2σ (I)] | R_1_ = 0.1461, wR_2_ = 0.3815 | | R_1_ = 0.0774, wR_2_ = 0.2245 | |  |
| Final R indexes [all data] | R_1_ = 0.1506, wR_2_ = 0.3863 | | R_1_ = 0.1395, wR_2_ = 0.2814 | |  |
| Largest diff. peak/hole / e Å^-3^ | 6.63/-8.56 | | 3.18/-1.52 | |  |

**Table S2.** Crystal data of Eu-MTB.

| Compunds | **Eu-MTB** |
| --- | --- |
| CCDC | 2175038 |
| Empirical formula | C_58_H_32_Eu_2_O_16_ |
| Formula weight | 1288.75 |
| Temperature/K | 301(2) |
| Crystal system | monoclinic |
| Space group | I2/m |
| a/Å | 11.6165(8) |
| b/Å | 17.0200(11) |
| c/Å | 23.4375(15) |
| α/° | 90 |
| β/° | 95.352(4) |
| γ/° | 90 |
| Volume/Å^3^ | 4613.7(5) |
| Z | 2 |
| ρ_calc_g/cm^3^ | 0.928 |
| μ/mm^‑1^ | 9.975 |
| F(000) | 1268.0 |
| Crystal size/mm^3^ | 0.9 × 0.5 × 0.3 |
| Radiation | CuKα (λ = 1.54178) |
| 2Θ range for data collection/° | 6.428 to 147.702 |
| Index ranges | -14 ≤ h ≤ 13, -20 ≤ k ≤ 19, -28 ≤ l ≤ 28 |
| Reflections collected | 20645 |
| Independent reflections | 4712 [R_int_ = 0.0578, R_sigma_ = 0.0444] |
| Data/restraints/parameters | 4712/0/183 |
| Goodness-of-fit on F^2^ | 1.077 |
| Final R indexes [I>=2σ (I)] | R_1_ = 0.0317, wR_2_ = 0.0859 |
| Final R indexes [all data] | R_1_ = 0.0321, wR_2_ = 0.0862 |
| Largest diff. peak/hole / e Å^-3^ | 0.91/-1.24 |

**Table S3.** Photophysical Parameters including Quantum yield (QY, Φ_F_) and average lifetime of ligands and RE-MOFs.

|  | Excitation  (nm) | Emission  (nm) | QY  (%) | Lifetime (ns) |
| --- | --- | --- | --- | --- |
| H_4_MTB powder | 395 | 465 | 0.99 |  |
| Eu-MTB | 335 | 432,615 | 16.58 |  |
| H_4_TCPP powder | 395 | 465 | 1.03 | 0.4657 |
| H_4_TCPP (70%H_2_O) | 395 | 465 | 9.43 |  |
| H_4_TCPP (DMF) | 395 | 465 | 2.47 |  |
| Eu-TCPP-1 | 377 | 615 | 19.28 |  |
| Tb-TCPP-1 | 390 | 425 | 6.87 | 0.5462 |
| Tb-TCPP-2 | 365 | 408 | 15.91 | 0.6099 |
| Y-TCPP-1 | 380 | 422 | 6.47 | 0.7117 |
| Y-TCPP-2 | 365 | 408 | 13.28 | 0.8484 |

**2.2-Figures**


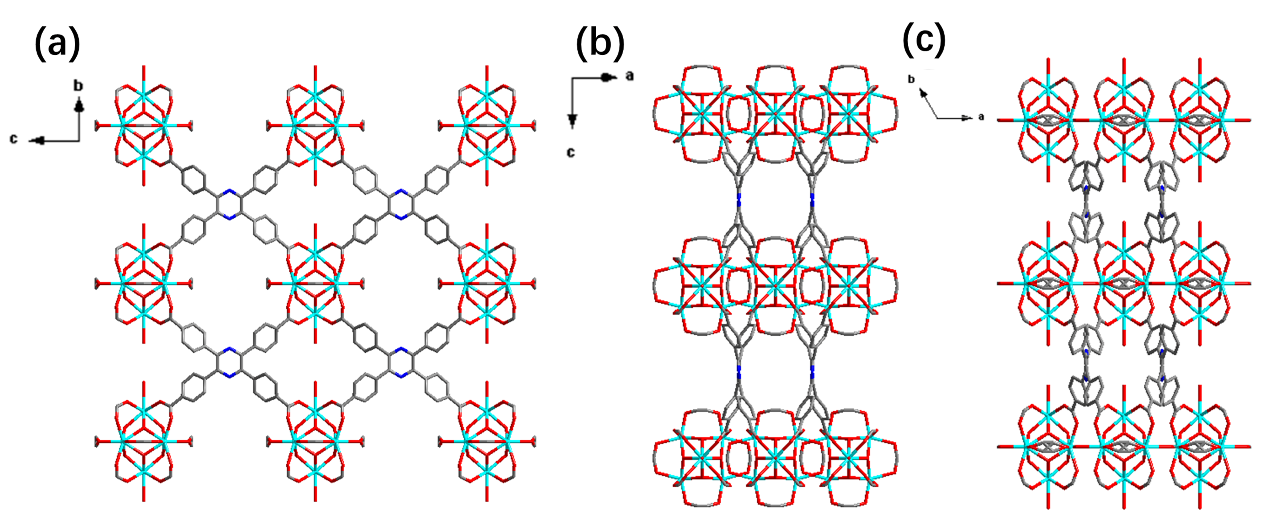


**Figure S1.** Three types of channels in Eu-TCPP-1 along the a, b, c axis direction.


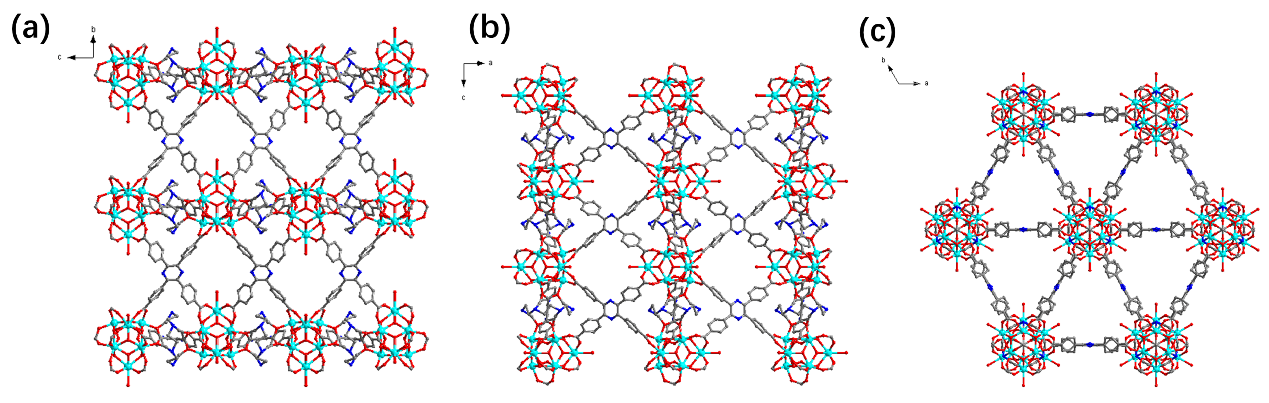


**Figure S2.** Three types of channels in Tb-TCPP-2 along the a, b, c axis direction.


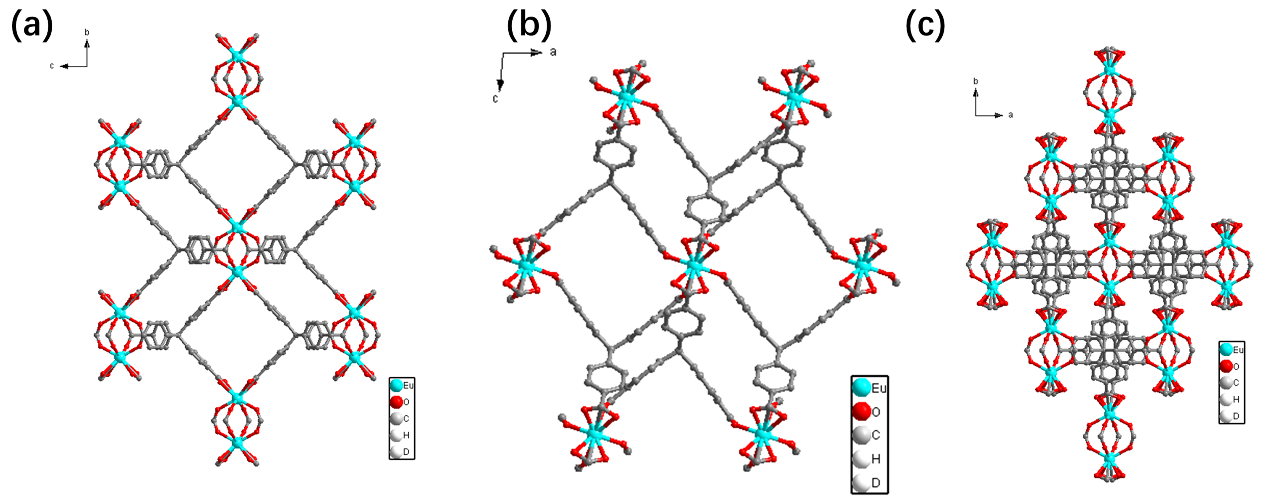


**Figure S3.** Three types of channels in Eu-MTB along the a, b, c axis direction.


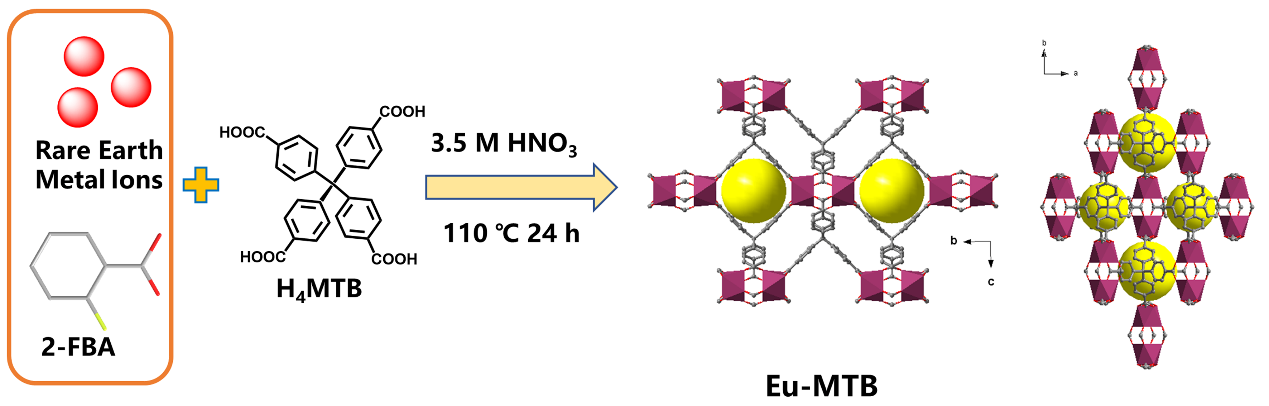


**Figure S4.** Schematic representation of the construction of Eu-MTB. H atoms and solvent molecules have been removed for clarity.


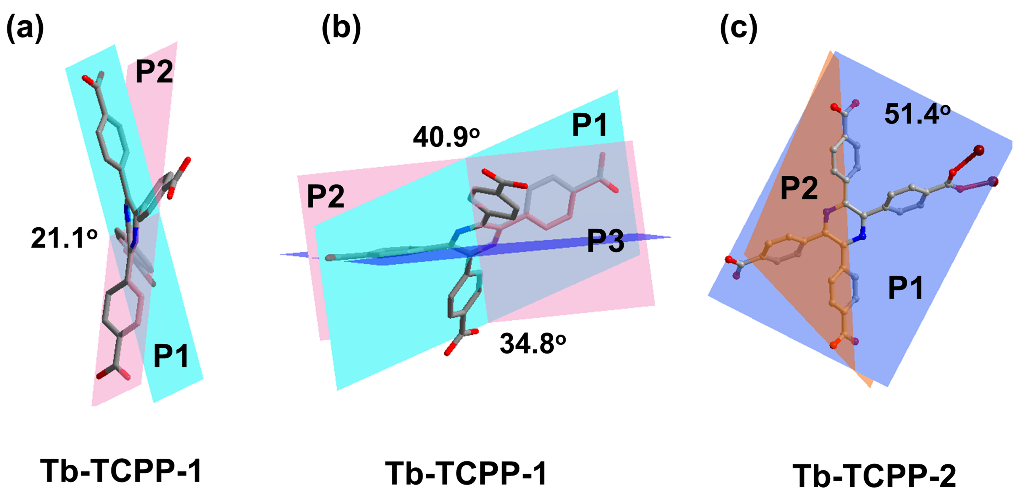


**Figure S5.** (a) The average of dihedral of P1-P2 plane is the twist pyrazine rings of the H_4_TCPP in Tb-TCPP-1. (b) P1-P2 is the average plane of the central pyrazine rings, P3 is the average plane of the phenyl rings adjacent to pyrazine rings of the H_4_TCPP in Tb-TCPP-1 optimized conformation, and the dihedral of P1-P3, P2-P3 are 34.8°, 40.9°, respectively; (c) P1 is the average plane of the central pyrazine rings. P2 is the average plane of the phenyl rings adjacent to pyrazine rings. All H atoms are omitted.


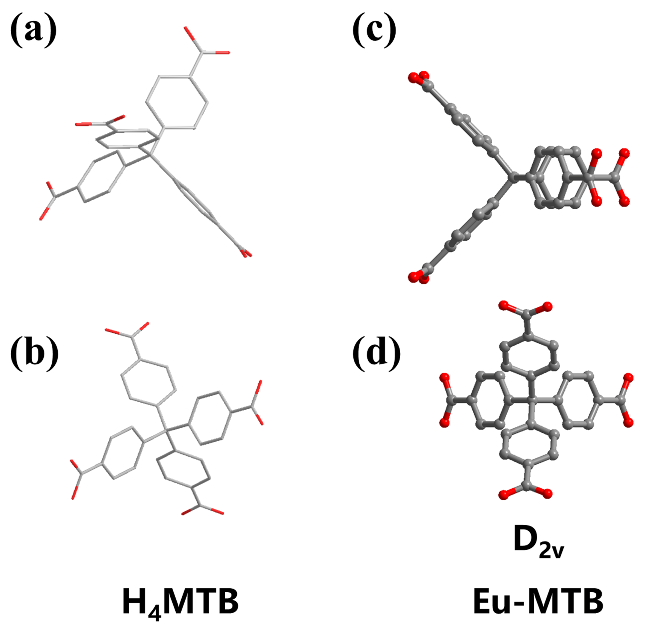


**Figure S6.** Conformations of the tetratopic ligands in Eu-MTB. (a, b) the free tetrahedral H_4_MB ligand. (c, d) Conformation of the tetrahedral ligand in the flu net framework. Black: C; red: O.

***Characterization***

To identify the phase purities of Eu-TCPP-1, Tb-TCPP-2 and Eu-MTB materials, powder X-ray diffraction (PXRD) were performed. As shown in Figures S7, PXRD patterns showed that the experimental materials matched well with their corresponding simulated ones. The purities of Tb-TCPP-1, Y-TCPP-2 and other isostructural were also confirmed (Figure S8-S10). According to the scanning electron microscopy (SEM) images and fluorescence photographs, Eu-TCPP-1 display a size about 100-120 *μ*m of regular octahedral morphology, and we observed a 40-50 *μ*m hexagonal prism crystals as for Tb-TCPP-2 (Figures S11, S16). Moreover, the Thermogravimetric analysis (TGA) curves shows that the decomposition temperature of Eu-TCPP-1 and Tb-TCPP-2 are around 550 °C under a nitrogen atmosphere. (Figure S12-S13). SEM and TGA also confirmed the stability of Eu-MTB (Figures S11, S14).


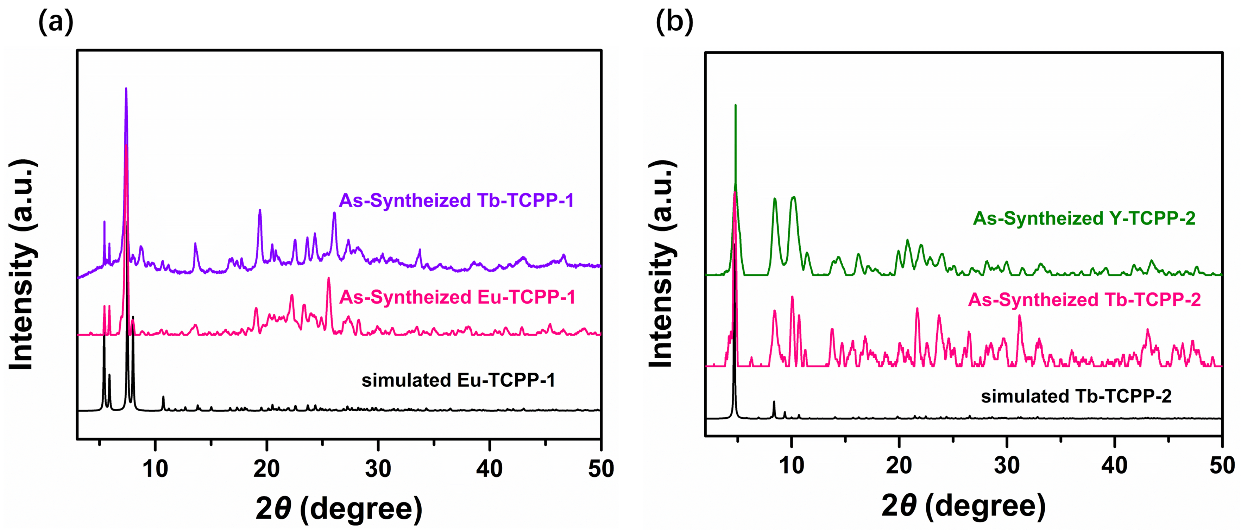


**Figure S7.** (a) PXRD patterns of synthesized Tb-TCPP-1 and Eu-TCPP-1, (b) Tb-TCPP-2 and Y-TCPP-2.

**Figure S8.** PXRD patterns of RE-TCPP-1 synthesized with different RE^3+^ (RE: Eu^3+^, Gd^3+^, Tb^3+^, Dy^3+^, Ho^3+^).

**Figure S9.** PXRD patterns of RE-MTB synthesized with different RE^3+^ (Eu^3+^, Tb^3+^).

**Figure S10.** PXRD patterns of RE-MTB synthesized with different RE^3+^ (RE: Sm^3+^, Eu^3+^, Gd^3+^, Tb^3+^, Dy^3+^, Ho^3+^, Er^3+^, Tm^3+^, Yb^3+^, Nd^3+^, Lu^3+^, La^3+^, Pr^3+^).

**
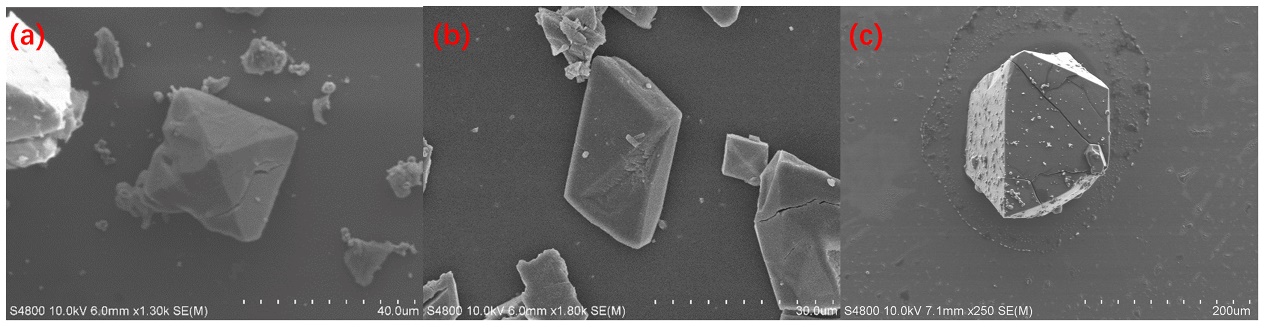
**

**Figure S11.** SEM images of (a) Eu-TCPP-1 and (b) Tb-TCPP-2, Eu-MTB.

**Figure S12.** TGA curves for Eu-TCPP-1 under nitrogen.

**Figure S13.** TGA curves for Tb-TCPP-2 under nitrogen.

**Figure S14.** TGA curves for Eu-MTB under nitrogen.

**Figure S15.** Luminescent spectra of H_4_TCPP powder.

**Photoluminescent properties of Eu-TCPP-1**

When excited at 377 nm, the solid-state Eu-TCPP-1 emit both ligand fluorescence and lanthanide fluorescence. The characteristic emissions at 589, 614, 653, and 701 nm are assigned to strong transitions ^5^D_0_ →7F_J_ (J = 1, 2, 3, and 4) of Eu^3+^ ions, and the weak emission bands at 417 nm are assigned to the H_4_TCPP ligand (Figure S15). The negligible emission band of ligands in the fluorescence spectrum of Eu-TCPP-1 demonstrates the high energy transfer (antenna effect) efficiency from H_4_TCPP to Eu^3+^, implying that the triplet energy level of H_4_TCPP matches the emitting level ^5^D_0_ of Eu^3+^ quite well. The single microcrystal of Eu-TCPP-1 emits bright red light when excited at 365 nm through the fluorescence microscope (the insets of Figure S16). Eu-TCPP-1 leading to the obvious red emission with quantum yield (Φ_F_) of 19.28% (Table S3).


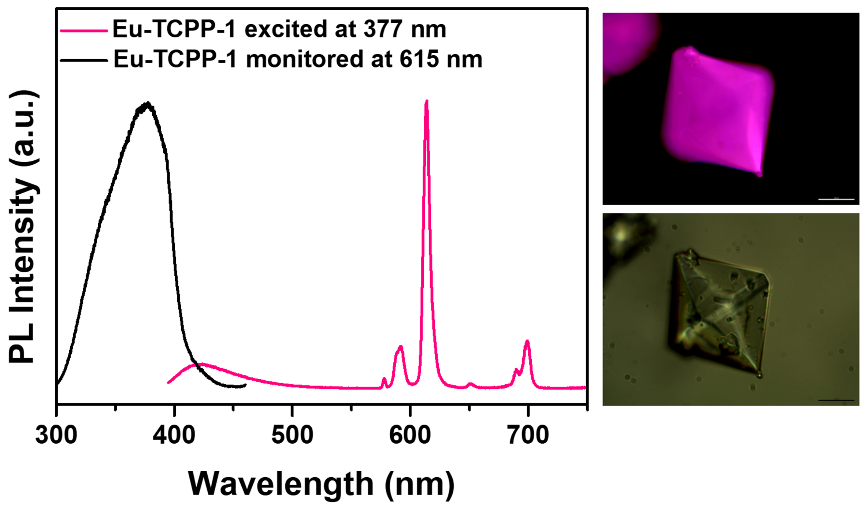


**Figure S16.** Emission spectra and crystal photographs of Eu-TCPP-1 excited at 377 nm. The crystal images are taken under 365 nm UV light. Scale bar, 20 μm.


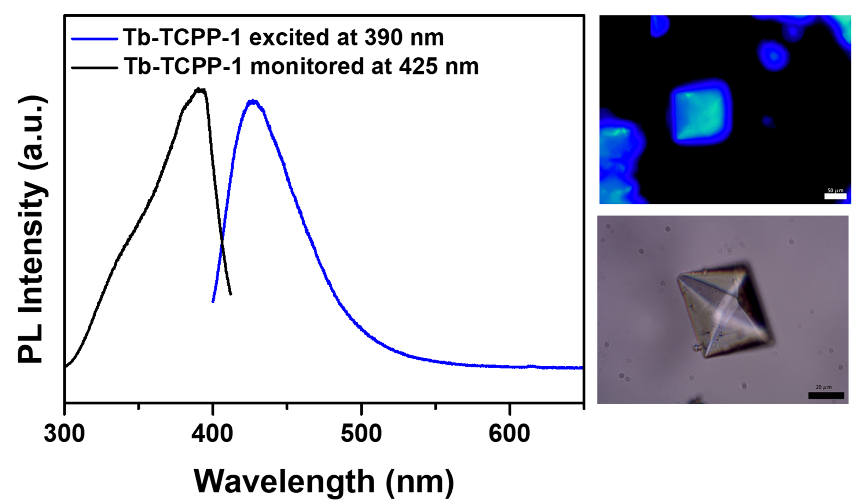


**Figure 17.** Emission spectra and crystal photographs of (a) Tb-TCPP-1 excited at 390 nm. Scale bar, 20 *μ*m, 50 *μ*m.


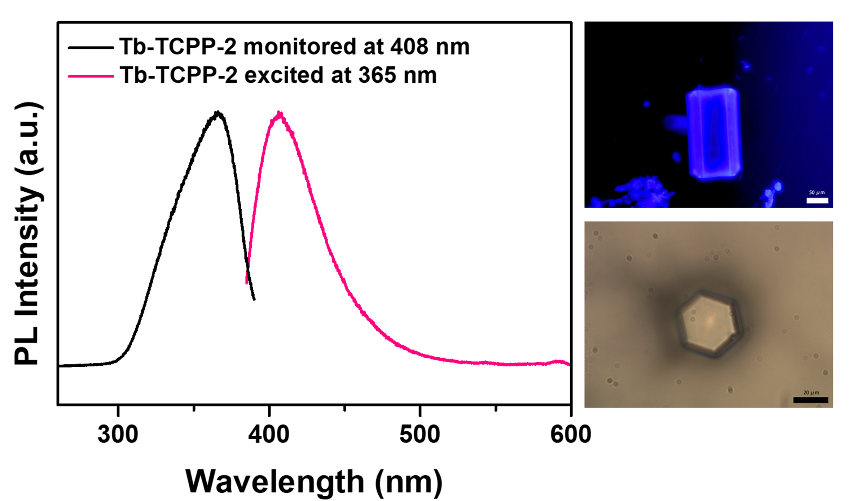


**Figure 18.** Emission spectra and crystal photographs of Tb-TCPP-2 excited at 365 nm; The crystal images are taken under 365 nm UV light. Scale bar, 20 *μ*m, 50 *μ*m.


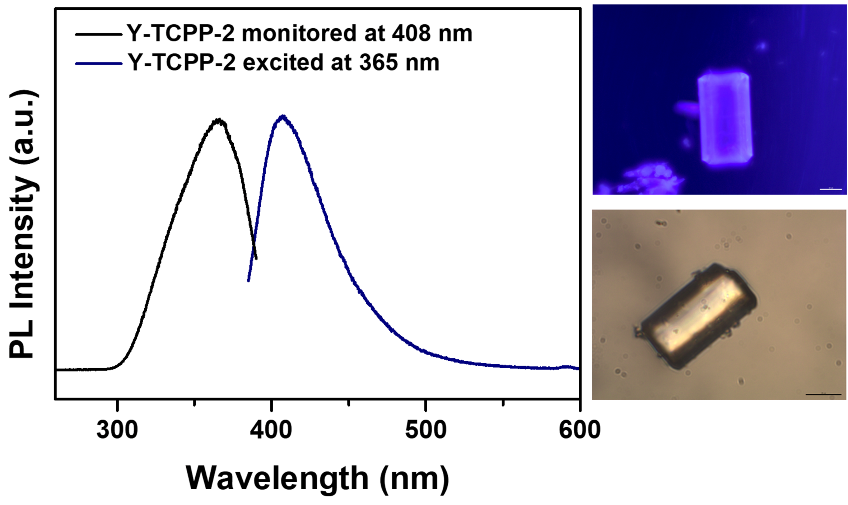


**Figure S19.** Emission spectra and crystal photographs of Y-TCPP-2 excited at 365 nm. The crystal images are taken under 365 nm UV light. Scale bar, 20 μm.

**Figure S20.** Emission spectra and crystal photographs of Y-TCPP-1 excited at 380 nm.

**Photoluminescent properties of Eu-MTB**

The photoluminescent (PL) properties of Eu-MTB and Tb-MTB were studied at room temperature (Figure S21). Excitation spectra of Eu-MTB and Tb-MTB both display an intense and broad band with a maximum at around 335 nm, which is presumably due to the π-π* electron transition of MTB^4-^ linkers (Figure S22). When excited at 335 nm, the solid-state Tb-MTB and Eu-MTB only emit lanthanide fluorescence. The characteristic emissions at 589, 614, 653, and 701 nm are ascribed to ^5^D_0_ →7F_J_ (J = 1, 2, 3, and 4) transitions of Eu^3+^ ions, and the strong emission bands at 488, 544, 583, and 621 nm, are assigned to the ^5^D_4_→^7^F_J_ (J = 6, 5, 4, and 3) transitions of Tb^3+^ ions, respectively (Figure S21). The single microcrystal of Eu-MTB and Tb-MTB emits bright red light and green light when excited at 365 nm through the fluorescence microscope (the insets of Figure 21). The ligand MTB^4-^ can sensitize Eu^3+^more efficiently than Tb^3+^, resulting in the emission of Eu^3+^ in Eu-MTB being stronger than Tb^3+^ in Tb-MTB. The Φ_F_ of Eu-MTB is 16.58% under the same measurement conditions (Table S3).


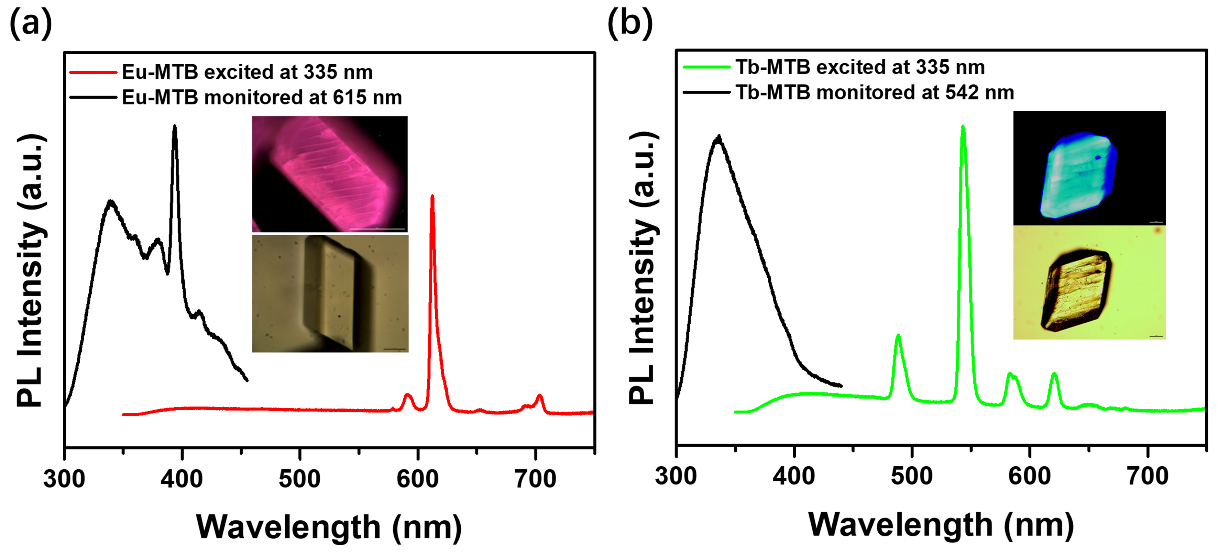


**Figure S21.** Emission spectra and crystal photographs of (a) Eu-MTB and (b) Tb-MTB excited at 335 nm. The crystal images are taken under 365 nm UV light. Scale bar, 20 μm.

**Figure S22.** Luminescent spectra H_4_MTB powder excited at 395 nm.

**Figure S23.** Emission spectra of Gd-MTB excited at 395 nm.

**AIE phenomenon of H_4_TCPP ligand**

To confirm the AIE phenomenon of H_4_TCPP ligand, the fluorescent property of H_4_TCPP ligand was first investigated in the DMF-H_2_O system. As shown in Figure S24, the emission of H_4_TCPP is very weak in pure DMF solution and increases remarkably until water fraction reaches 70%, afterward, the emission intensity decreases. Since water is a poor solvent for H_4_TCPP, the addition of water will lead to the formation of nanoaggregates of H_4_TCPP, resulting in the AIE effect of H_4_TCPP. The emission intensity decreases at high water fraction (>70%) may be due to the presence of four carboxylic acid groups of H_4_TCPP. The AIE feature of H_4_TCPP was evidenced by a test in quantum yield in DMF solution (Φ_F_ = 2.47%) and in the DMF/H_2_O=30%/70% (Φ_F_ = 9.43%) (Table S3). Thus, H_4_TCPP feature the unique aggregation-enhanced emission (AEE) characteristics.


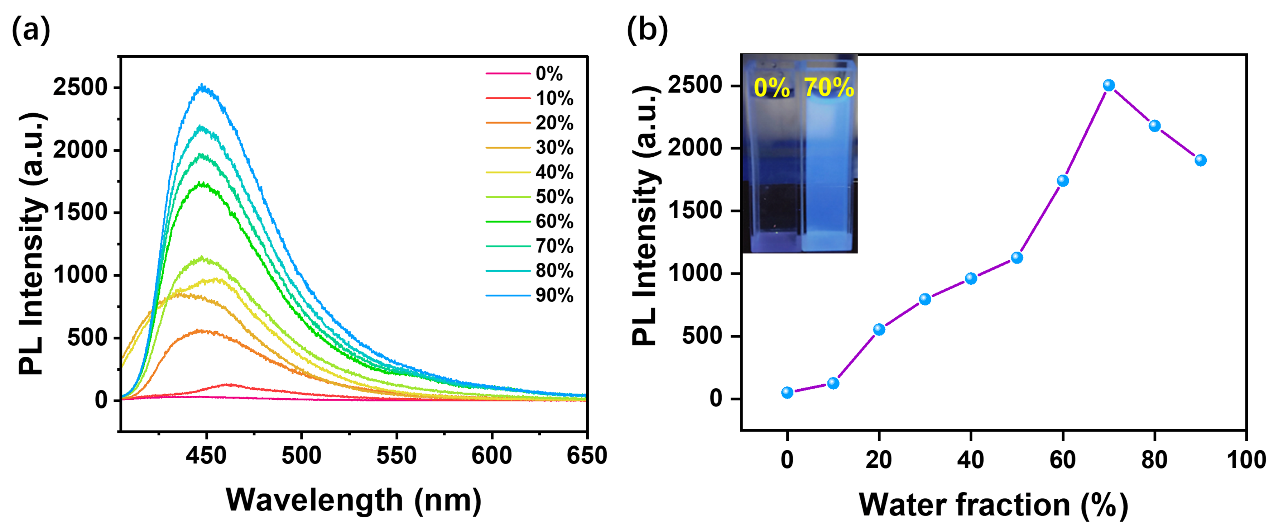


**Figure S24.** (a) Fluorescence spectra of H_4_TCPP in DMF-water mixtures with different water fractions. (b) Plot of PL intensity versus the composition of DMF-water mixture of H_4_TCPP (inset: the fluorescence photograph of H_4_TCPP in DMF-water mixtures with different water fractions taken under irradiation of 365 nm UV light).

**Figure S25.** The excitation and emission spectra of H_4_TCPP, Tb-TCPP-1 and Tb-TCPP-2 at room temperature (RT).

**Figure S26.** The emission spectra of Tb-TCPP-1, Tb-TCPP-2 and H_4_TCPP in solid state.

**Figure 27.** Temperature-dependent emission spectrum of Tb-TCPP-2 cooled with liquid nitrogen. *λ*_ex_ = 365 nm.

**Figure S28.** The lifetime of H_4_TCPP, Y-TCPP-1 and Y-TCPP-2 excited at 375 nm.


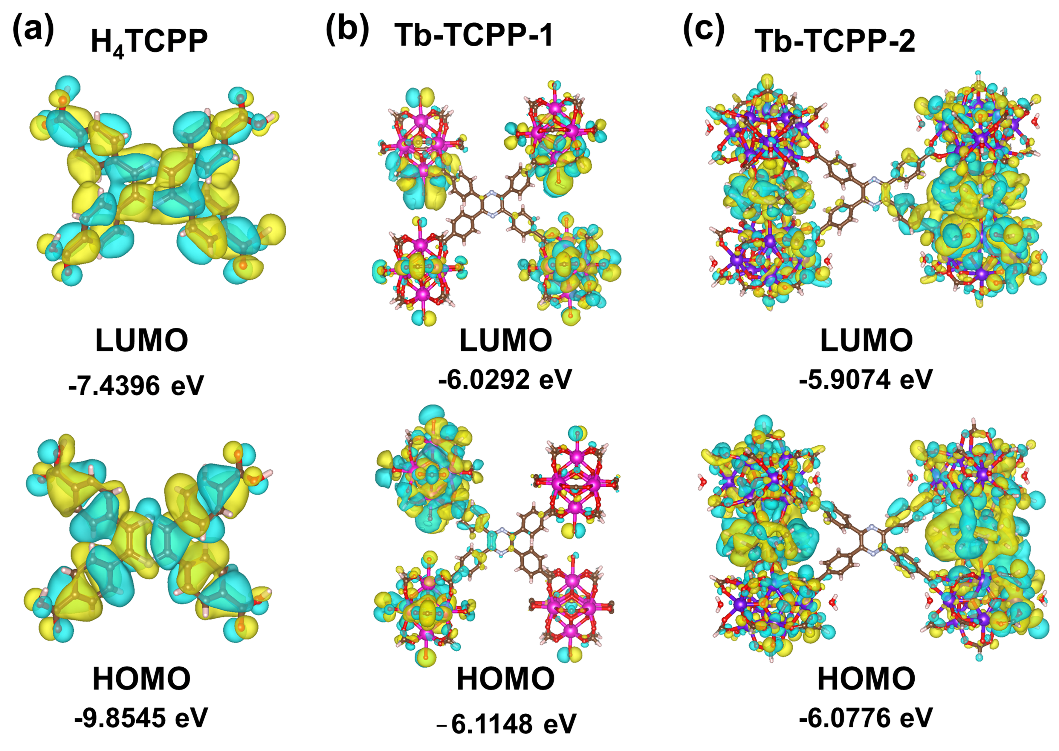


**Figure S29.** Contour plots of the HOMO and LUMO for H_4_TCPP, Tb-TCPP-1 and Tb-TCPP-2.

**Figure S30.** Fluorescence lifetime measurements for a powder sample of H_4_TCPP using an excitation of 375 nm and an emission of 465 nm.

**Figure S31.** Fluorescence lifetime measurements for a powder sample of Tb-TCPP-1 using an excitation of 375 nm and an emission of 415 nm.

**Figure S32.** Fluorescence lifetime measurements for a powder sample of Tb-TCPP-2 using an excitation of 375 nm and an emission of 408 nm.


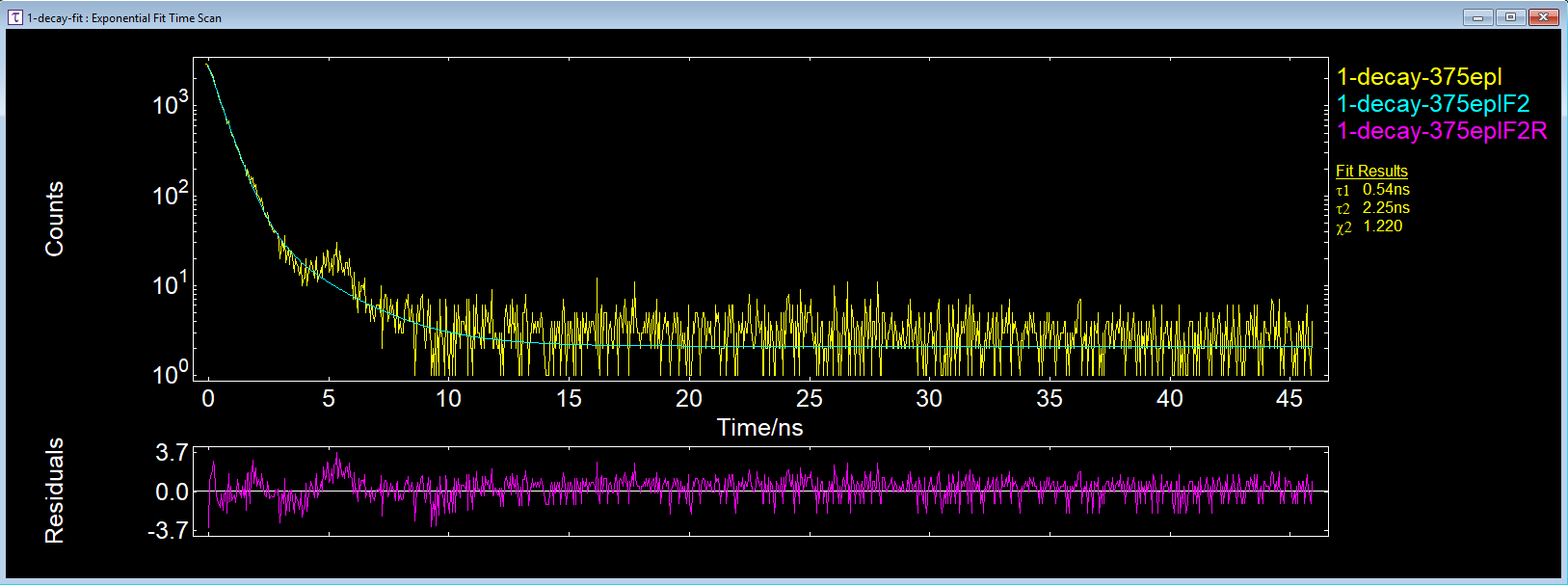


**Figure S33.** Fluorescence lifetime measurements for a powder sample of Y-TCPP-1 using an excitation of 375 nm and an emission of 422 nm.


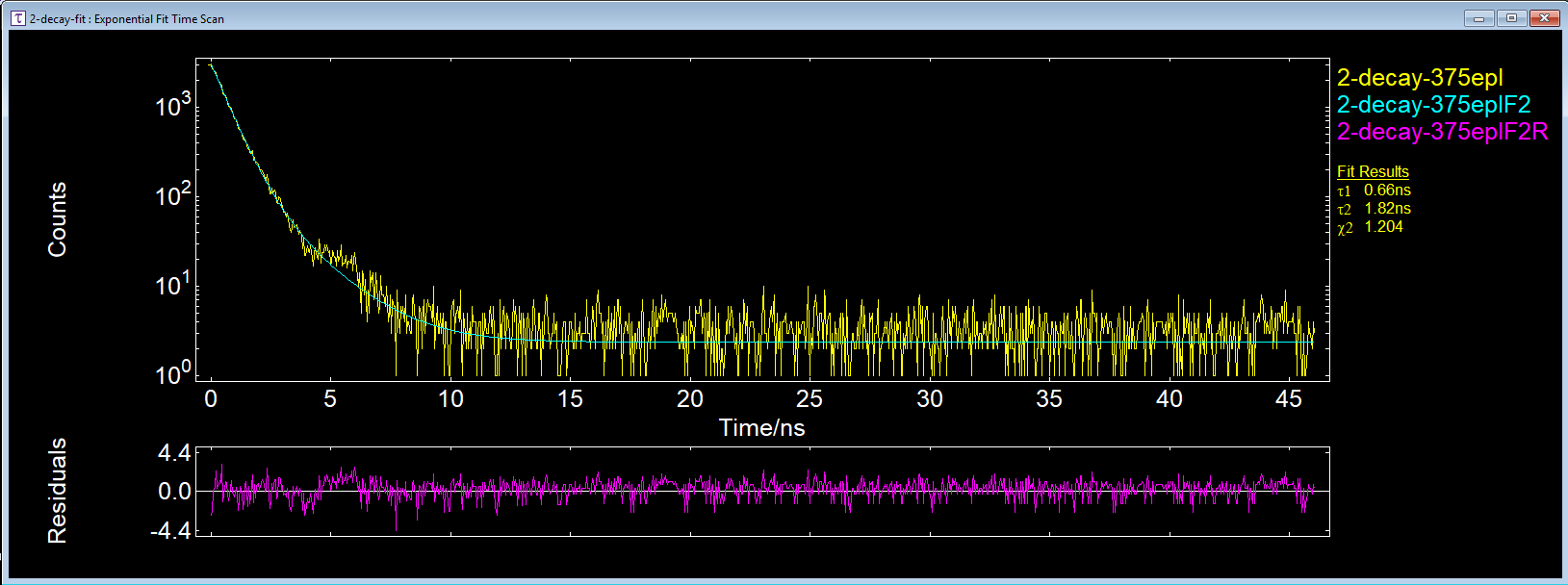


**Figure S34.** Fluorescence lifetime measurements for a powder sample of Y-TCPP-2 using an excitation of 375 nm and an emission of 408 nm.

**REFERENCE**

(1) Kresse, G.; Furthmuller, J., "Efficient Iterative Schemes for ab initio Total-energy Calculations Using a Plane-wave Basis Set," *Physical Review B*, vol. 54, no. 16, pp. 11169, 1996.

(2) Perdew, J. P.; Burke, K.; Ernzerhof, M., "Generalized Gradient Approximation Made Simple," *Physical Review Letters*, vol. 77, no. 18, pp. 3865, 1996.

(3) Hammer, B.; Hansen, L. B.; Norskov, J. K., "Improved Adsorption Energetics within Density-functional Theory Using Revised Perdew-Burke-Ernzerhof Functionals," *Physical Review B*, vol. 59, no. 11, pp. 7413, 1999.
